# S100A8 inhibits PDGF-induced proliferation of airway smooth muscle cells dependent on the receptor for advanced glycation end-products

**DOI:** 10.1186/s40659-017-0128-5

**Published:** 2017-06-21

**Authors:** Yu-Dong Xu, Yu Wang, Lei-Miao Yin, Ling-Ling Peng, Gyoung-Hee Park, Yong-Qing Yang

**Affiliations:** Laboratory of Molecular Biology, Shanghai Research Institute of Acupuncture and Meridian, Yueyang Hospital of Integrated Traditional Chinese and Western Medicine, Shanghai University of Traditional Chinese Medicine, 650 South Wanping Road, Shanghai, 200030 China

**Keywords:** S100A8, Airway smooth muscle cells, Cell proliferation, The receptor for advanced glycation end-products, Platelet-derived growth factor

## Abstract

**Background:**

Airway remodeling is a key feature of asthma, characterized by increased proliferation of airway smooth muscle cells (ASMCs). S100A8 is a calcium-binding protein with a potential to regulate cell proliferation. Here, the effect of exogenous S100A8 protein on the proliferation of ASMCs induced by platelet-derived growth factor (PDGF) and the underlying molecular mechanism was investigated.

**Methods:**

Rat ASMCs were cultured with or without a neutralizing antibody to the receptor for advanced glycation end-products (RAGE), a potential receptor for S100A8 protein. Purified recombinant rat S100A8 protein was then added into the cultured cells, and the proliferation of ASMCs induced by PDGF was detected by colorimetric-based WST-8 assay and ampedance-based xCELLigence proliferation assay. The expression levels of RAGE in ASMCs were analyzed using western blotting assay.

**Results:**

Results showed that exogenous S100A8 inhibited the PDGF-induced proliferation of rat ASMCs in a dose-dependent manner with the maximal effect at 1 μg/ml in vitro. Furthermore, when ASMCs was pre-treated with anti-RAGE neutralizing antibody, the inhibitory effect of S100A8 on PDGF-induced proliferation was significantly suppressed. In addition, neither the treatment with S100A8 or PDGF alone nor the pre-treatment with rS100A8 followed by PDGF stimulation affected the expression levels of RAGE.

**Conclusions:**

Our study demonstrated that S100A8 inhibits PDGF-induced ASMCs proliferation in a manner dependent on membrane receptor RAGE.

## Background

Airway remodeling is a hallmark pathological feature of chronic inflammatory airway diseases including asthma, chronic obstructive pulmonary disease and other types of bronchiectasis. It is often considered the result of longstanding airway inflammation and ultimately leads to narrowing of the bronchial lumen and progressive loss of lung function [1]. The abnormal proliferation of airway smooth muscle cells (ASMCs) is believed to be a major contributor to the pathological process of airway remodeling [2]. An increased proliferation of ASMCs has been shown in both asthmatic patients and animal models of allergic airway inflammation [3, 4]. Many inflammatory mediators are released from inflammatory and structural cells in the asthmatic airways, and some can induce airway smooth muscle mitogenesis in vitro. Growth factors such as platelet-derived growth factor (PDGF), epidermal growth factor (EGF), basic fibroblast growth factor have been identified as the main mitogens that modulate the ASMCs growth [5]. The protective mechanisms that prevent or attenuate the growth factor-induced proliferation of ASMCs are feasible therapeutic targets for the treatment of inflammatory airway diseases.

S100A8 (also known as MRP8 and calgranulin A), an important member of the S100 protein family, is a low-molecular-weight (10.8 kDa) calcium-binding protein containing conserved EF-hand structural motifs [6]. Our previous study showed that the S100A8 protein is differentially expressed in the airways of asthmatic rat model and may be associated with the pathophysiological process of asthma [7]. An ambivalent role for S100A8 protein in inflammatory diseases has been documented, and evidence indicates that the exact extracellular function of S100A8 protein depends on the local tissue environment to which it is released [8, 9]. We have confirmed that exogenous S100A8 protein can inhibit PDGF-induced migration of ASMCs in vitro [10]. However, the effects and mechanisms of S100A8 on ASMCs proliferation remain unclear.

It has been reported that exogenous S100A9 protein, the dimerization partner of S100A8, promoted the proliferation and growth of human hepatocellular carcinoma cells in vitro and in vivo [11]. However, the over-expression of intracellular S100A8/A9 was shown to reduce the proliferation of HaCaT keratinocytes [12]. It is necessary to elucidate the potential role of S100A8 protein in the proliferation of ASMCs associated with airway remodeling. Therefore, the aim of our present study was to investigate the effects of exogenous S100A8 on PDGF-induced excessive proliferation of primary cultured ASMCs, and study the possible molecular mechanism.

## Methods

### Cell culture

The primary rat ASMCs were dissected, purified, and cultured in Dulbecco’s modified Eagle’s medium (DMEM; HycloneLabs., Logan, UT) containing 10% fetal bovine serum (FBS; Gibco, Grand Island, NY), 100 U/ml penicillin, and 0.1 mg/ml streptomycin (Hyclone Labs., Logan, UT) as described previously [10]. Cultures were maintained at 37 °C under an atmosphere containing 5% CO_2_ (Forma Scientific) and the medium was replaced with fresh medium every 48 h. ASMCs were identified by positive immunocytochemical staining with alpha-smooth muscle actin. All experiments were performed on cultured cells between passages 3 and 6.

### Recombinant protein preparation

The PET-22b-rS100A8 plasmid used in the current study has been described previously [10]. Briefly, PET-22b-rS100A8 was cloned through the *Nde*I and *Xho*I restriction sites and were transformed into BL21 (DE3) by calcium chloride transformation. The positive *Escherichia coli* strain containing the recombinant pET-22b-rS100A8 plasmid was induced by the addition of isopropyl-β-d-thiogalactopyranoside (IPTG). The bacteria cells were then collected and sonicated on ice, and centrifuged at 4 °C. The recombinant rat S100A8 protein (rS100A8) in the supernatant was purified by affinity chromatography purification using Ni Sepharose 6 Fast Flow beads (GE Healthcare, Uppsala, Sweden) and by ion-exchange chromatography purification using Q Sepharose Fast Flow beads (GE Healthcare, Uppsala, Sweden) by gravity flow according to the manufacturer’s instructions. A western blotting assay was conducted to analyze the antigenic activity of rS100A8 protein.

### Colorimetric-based proliferation assay

Colorimetric-based cell proliferation assay was performed using Cell Counting Kit-8 (CCK-8; Dojindo, Kumamoto, Japan), which is based on bioreduction of 2-(2-methoxy-4-nitrophenyl)-3-(4-nitrophenyl)-5-(2,4-disulfophenyl)-2H-tetrazolium (WST-8) that produces a water-soluble formazan dye. Cells were seeded at a density of 3000 cells/well in a 96-well plate and were allowed to attach for 24 h in DMEM medium containing 10% FBS. Then cells were starved in serum-free medium overnight and were preincubated with or without rS100A8 (at 50 ng/ml, 100 ng/ml, 250 ng/ml, 500 ng/ml, 1 μg/ml, 2.5 μg/ml and 5 μg/ml) for 1 h before stimulation with PDGF (20 ng/ml) for 24, 48, and 72 h. Hydrocortisone at the concentration of 20 μg/ml served as a positive control. At the end of the each cultural time point, cells were incubated with 10 μl prepackaged CCK-8 solution for 3 h at 37 °C. After incubation, absorbance at 450 nm was measured using Synergy H1 Hybrid microplate reader (Biotek Instruments, Winooski, VT, USA) with a reference wavelength of 600 nm. For the RAGE blockade analysis, cells were pretreated with an anti-rat RAGE extracellular domain monoclonal antibody (5 μg/ml, R&D Systems, Minneapolis, MN) or control IgG (Isotype Control-Goat IgG (Fc), Abcam, Cambridge, UK) for 30 min before the addition of rS100A8 (1 μg/ml) and PDGF, and the cell proliferation was detected at the 72 h time point. Each condition was done in quadruplicate, and the experiment was repeated 3 times.

### Impedance-based xCELLigence proliferation assay

The xCELLigence system (Roche Applied Science) provides a noninvasive and label-free monitoring on the cell viability/proliferation based on the measurement of electrical impedance for analysis the response of cultured cells in real-time. Increased impedance is resulted if cells adhere and proliferate on the surface of electrodes as biological cells are very poor conductors at low frequencies [13]. For xCELLigence proliferation assay, the background impedance of culture medium in E-plate16 (Roche Diagnostics) was measured firstly. Then cells were seeded into the E-plate 16 at a density of 3000 cells/well and allowed to attach for 3 h in 200 ml DMEM medium containing 1% FBS in the absence or presence of rS100A8 (at 500 ng/ml, 1 μg/ml and 2.5 μg/ml). Hydrocortisone at the concentration of 20 μg/ml served as a positive control. After 30 min, PDGF was added to the culture medium at a final concentration of 20 ng/ml. For the RAGE blockade analysis, cells were pretreated with an anti-rat RAGE extracellular domain monoclonal antibody (5 μg/ml) or control IgG (5 μg/ml, Isotype Control-Goat IgG (Fc), Abcam, Cambridge, UK) for 30 min before the addition of rS100A8 (1 μg/ml) and PDGF. Each condition was performed in triplicate, and the impedance signal (termed as “cell index” in manufacturer’s software) was recorded every 30 min for a total of 72 h. Cell-doubling time was calculated using cell index data with the aid of Doubling Time software (http://www.doubling-time.com).

#### Protein extraction and western blotting

Cells were harvested after the treatment with PDGF and/or S100A8 protein, and the membrane protein fraction was isolated using Transmembrane Protein Extraction Kit (Novagen, Madison, WI) according to the manufacturer’s instructions. Briefly, cells were permeabilized using Extraction Buffer 1 and soluble cytoplasmic fraction separated from insoluble membrane fraction by centrifugation. The pellet was then resuspended and membrane proteins were extracted from the lipid bilayer using Extraction Buffer 2B. Samples containing 50 μg of denatured total proteins were separated by SDS-PAGE using 12% polyacrylamide gels, and then electrotransferred onto polyvinylidene fluoride membranes (Millipore; Billerica, MA). After blocking with PBS containing 5% nonfat milk, the blotted membranes were probed overnight at 4 °C with specific primary antibody against RAGE (0.1 μg/ml, R&D). Immunoreactions were detected using HRP-conjugated secondary antibody and enhanced chemiluminescent analysis system. Determination of band densities was performed using Image J software (NIH, Bethesda, MD) and was normalized to control levels.

### Statistical analysis

Data are presented as the mean values ± standard deviation. The differences were analyzed using one-way ANOVA followed by the least significant difference (LSD) test. A value of *P* < 0.05 was considered to represent a statistically significant difference.

## Results

### Purification of recombinant S100A8 protein

**Fig. 1:**
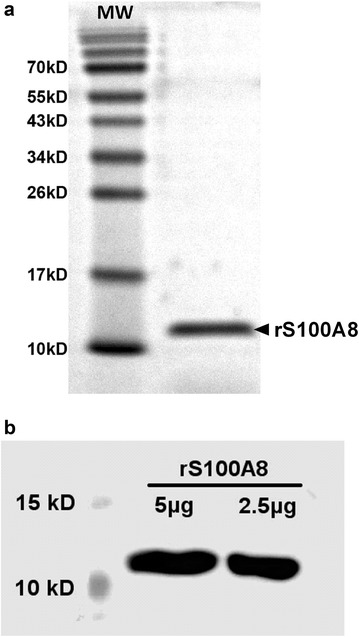
The purification of recombinant rat S100A8 protein (rS100A8). **a** SDS-PAGE analysis of the recombinant rS100A8 that was purified with affinity chromatography and ion exchange chromatography. **b** Western blot analysis showed the antigenic activity of rS100A8 protein

SDS-PAGE analysis showed that rS100A8 protein was achieved with higher than 95% purity after the affinity chromatography purification (Fig. 1a). Western blotting assay with a specific antibody against rat S100A8 (1:3000, Santa Cruz sc-8113) demonstrated the antigenic activity of rS100A8 protein (Fig. 1b).

### rS100A8 inhibits the PDGF-induced proliferation of ASMCs

**Fig. 2:**
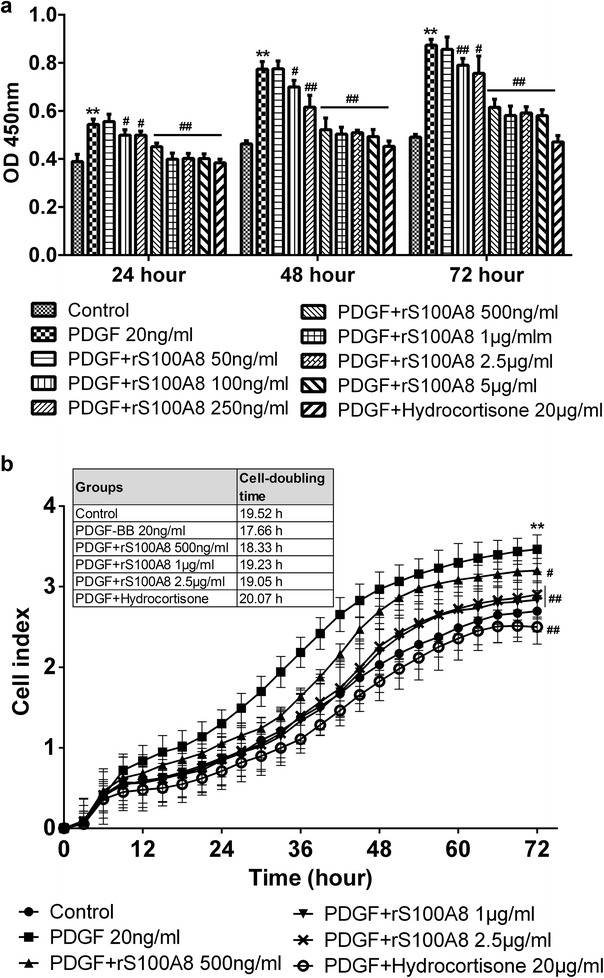
Exogenous S100A8 protein inhibited PDGF-induced proliferation in ASMCs. **a** ASMCs proliferation was measured using colorimetric-based proliferation assay. **b** Real-time cell proliferation was measured using the impedance-based xCELLigence proliferation assay, for which the statistical analysis was performed at 72 h time point. Data are expressed as mean ± SD derived from three different set of experiments; **P < 0.01 compared with control; ^#^P < 0.05, ^##^P < 0.01 compared with PDGF group. The *inset table* presents cell-doubling times of each group

The effect of different doses of rS100A8 (50 ng/ml, 100 ng/ml, 250 ng/ml, 500 ng/ml, 1 μg/ml, 2.5 μg/ml and 5 μg/ml) on PDGF-induced ASMC proliferation was firstly assessed using the Colorimetric-based proliferation (CCK-8) assay. As anticipated, a significant increase in the proliferation of ASMCs was induced by 20 ng/ml of PDGF, which reached to 1.4-, 1.7-, 1.8-folds of the control group at the point of 24, 48 and 72 h (all *P* < 0.01). As the positive control, hydrocortisone at the concentration of 20 μg/ml completely blocked this process. The hyperproliferative effect of PDGF was significantly suppressed in a dose-dependent manner by preincubation with rS100A8. As shown in Fig. 2a, rS100A8 at 1 μg/ml produced the maximal inhibitory effect on PDGF-induced proliferation in ASMCs (about 73, 65 and 66% of the PDGF group at the point of 24, 48 and 72 h; all *P* < 0.01).

As an alternative measurement of cell proliferation, the xCELLigence real-time cell analyzer system was used to confirm the inhibitory effect of rS100A8 at 500 ng/ml, 1 μg/ml and 2.5 μg/ml on PDGF-induced ASMCs proliferation. It was shown that rS100A8 can inhibit the proliferation of ASMCs over a time period of 72 h with the maximal effect at 36 h after the stimulation of PDGF (about 61% of the PDGF group at 1 μg/ml; *P* < 0.01, Fig. 2b). In addition, ASMCs stimulated with PDGF displayed a shorter cell-doubling time of 17.66 h as compared with controls (19.52 h), whereas cells pretreated with 1 μg/ml of rS100A8 displayed increased cell-doubling time of 19.23 h (Fig. 2b). Together, these results indicate that exogenous S100A8 can lead to a decreased cell growth rate and inhibit the PDGF-induced proliferation of ASMCs in vitro.

### Blockade of RAGE counteracts the inhibitory effect of rS100A8 on PDGF-induced ASMCs proliferation

**Fig. 3:**
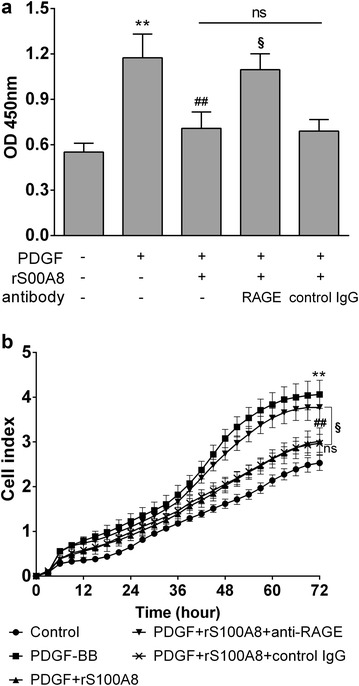
Blockade of RAGE suppressed the inhibitory effect of rS100A8 on PDGF-induced proliferation in ASMCs. **a** ASMCs proliferation was measured at the 72 h time point after PDGF stimulation using colorimetric-based proliferation assay. **b** Real-time cell proliferation was measured using the impedance-based xCELLigence proliferation assay, for which the statistical analysis was performed at the last time point. Means for three replicates ± SD are shown; **P < 0.01, control vs PDGF group; ^##^P < 0.01, PDGF group vs PDGF + rS100A8 group; ^§^P < 0.01, PDGF + rS100A8 group vs PDGF + rS100A8 + RAGE antibody group; ns (non-significant), PDGF + rS100A8 + RAGE antibody group vs PDGF + rS100A8 + control IgG group

The inhibitory effect of rS100A8 (1 μg/ml) on PDGF-induced ASMCs proliferation was reexamined after blocking RAGE, which is known to be a potential receptor of S100A8. An anti-rat RAGE extracellular domain monoclonal antibody was used to block the extracellular binding sites of RAGE for ligand engagement. As shown in Fig. 3, the two different proliferation assays both showed that the blockade of the cell membrane receptor RAGE with specific antibody significantly weakened the inhibitory effect of rS100A8 on PDGF-induced proliferation of ASMCs, whereas the non-specific control IgG had no effect on rS100A8 inhibiting PDGF-induced cell proliferation. Additionally, the proliferation of control cells was not affected by the sole addition of the RAGE antibody (data not shown).

### rS100A8 treatment has no effect on the expression level of RAGE

**Fig. 4:**
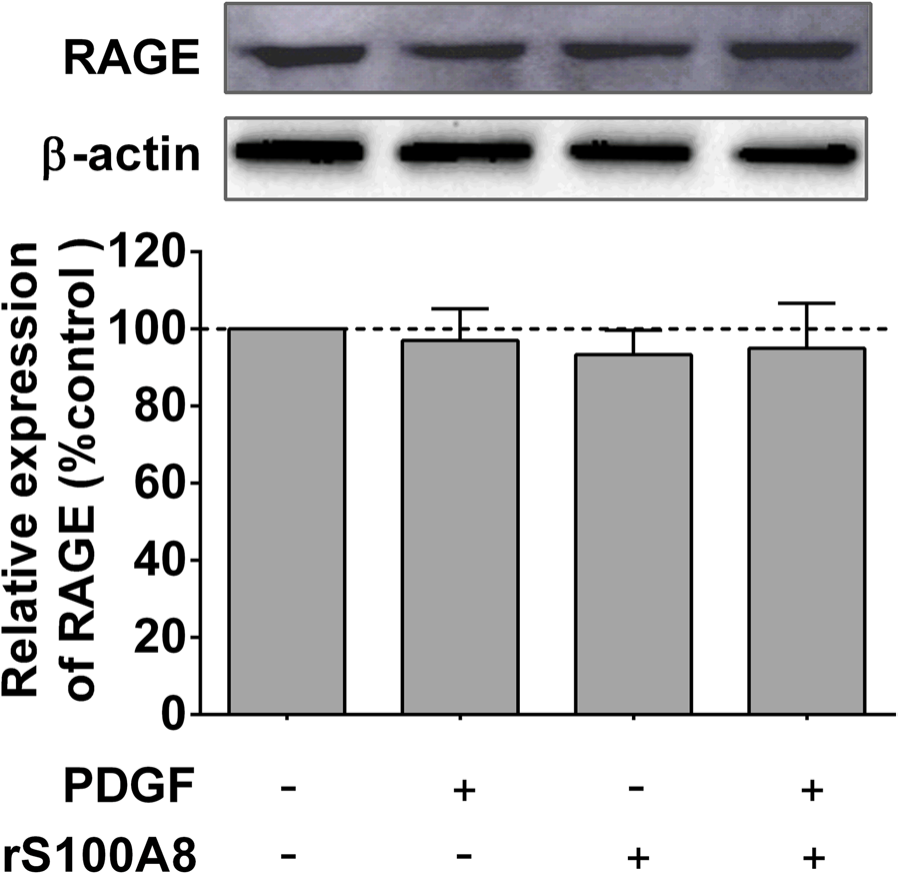
The expression level of RAGE was not affected by PDGF or rS100A8 treatment. Expression levels of RAGE in PDGF-stimulated ASMCs that were pretreated with or without rS100A8 (1 μg/ml) were detected by western blotting. A representative immunoblot is shown on the *top of the figure*. Data are presented as the mean ± SD of three separate experiments

We further performed western blotting assay to analyze whether rS100A8 can change the expression of RAGE in ASMCs. As shown in Fig. 4, neither the treatment with rS100A8 or PDGF alone nor the pre-treatment with rS100A8 followed by PDGF stimulation affected the expression levels of RAGE as compared to untreated control cells, suggesting that rS100A8 protects against PDGF-induced proliferation of ASMCs possibly through modulation of a RAGE-dependent pathway rather than changing the expression level of RAGE itself.

## Discussion

Airway remodeling is a prominent pathophysiologic feature of asthma and it is related to the severity of the disease; however, the therapeutic strategy directed to this event has not been well established. In the present study, we showed that exogenous S100A8 protein significantly inhibited PDGF-BB induced ASMCs proliferation via the membrane receptor RAGE. These observations are important for developing novel therapeutic strategies against asthma, because hyperproliferation of ASMCs contributes to the airway remodeling that leads to increased airway hyperresponsiveness and irreversible airway obstruction, particularly in refractory asthma.

Accumulating evidence has demonstrated that the S100A8 protein, which is mainly secreted by neutrophils, monocytes and macrophages, has both intracellular and extracellular roles [6]. Although S100A8 and S100A9 always exist as homodimers, S100A8 has been found to be the active component of the S100A8/A9 complex and that S100A9 predominantly functions as a regulatory subunit [14]. Several in vitro studies have suggested the role of endogenous S100A8/A9 in some intracellular homeostatic processes including calcium sensing, activation of NADPH oxidase and arachidonic acid transport [6, 15]. The secreted S100A8 protein was reported to act as an extracellular damage-associated molecular pattern that activating inflammatory signaling cascades and triggering cellular responses through interaction with cell surface pattern recognition receptors such as Toll like receptors and RAGE [16]. However, there is still controversy regarding the functional role of S100A8 and its dimerization partner S100A9 in the process of cell proliferation. Recent experimental studies showed that exogenously added S100A8 and S100A9 proteins enhanced the proliferation of tumor cells such as breast cancer cells, colorectal and pancreatic cancer cells [17, 18]; the overexpression of intracellular S100A8 protein had a growth-promoting activity in keratinocytes [19]. In contrast, some of past studies showed that S100A8 and S100A9 protein have inhibitory effects on the proliferation of carcinoma cells and normal fibroblasts by inducing cell apoptosis [20]. Our data also suggests that exogenous S100A8 protein at 1 μg/ml significantly suppresses the cell proliferation induced by PDGF in ASMCs, which is consistent with the observation that S100A8/A9 over-expression leads to reduced proliferation in HaCaT keratinocytes [12]. The reason for the discrepancy between the results mentioned above is unclear but may relate to the active form of the protein and the pathophysiological status of the targeted cells.

PDGF-BB is known as one of the most potent mitogens for ASMCs and plays an important role during ASMCs proliferation in developing airway remodeling [1]. In asthmatic airways, PDGF can be secreted by a variety of cell types including the damaged epithelial cells, macrophages, fibroblasts, and many other pro-inflammatory cells [21]. Both in vivo and in vitro studies have demonstrated that PDGF promoted the pro-remodeling function of ASMCs by inducing the cell proliferation and migration [22, 23]. Therefore, we used PDGF as an in vitro mitogen to study the possible effect of S100A8 on the proliferation of ASMCs. Cells stimulated by PDGF generate endogenously and constitutively reactive oxygen species (ROS), which are utilized in the induction and maintenance of signal transduction pathways involved in cell growth and differentiation [24, 25]. Evidence showed that pretreatment with the antioxidant *N*-acetyl-cysteine significantly attenuated PDGF-induced proliferation in vascular smooth muscle cells [26]. It is noteworthy that S100A8 has potent oxidant-scavenging activity [8, 27]. The oxidant-scavenging activity of S100A8 protein has been further supported by a recent study finding that S100A8 inhibits both IgE-mediated MC degranulation and the production of some pro-inflammatory cytokines by scavenging ROS generated by activated leukocytes [28]. These observations provide a potential link between oxidant-scavenging activity of S100A8 and its inhibitory effect on the PDGF-induced proliferation of ASMCs.

RAGE has been proposed to serve as a cell surface receptor for S100 proteins and mediate most of cellular responses through mitogen-activated protein kinase/extracellular-signal-regulated kinase (MAPK/ERK) and NF-κB signaling pathways [29]. Recent studies provide more direct evidence for interaction of S100A8 to RAGE in a variety of cell lines [18, 30]. The S100A8 protein has been reported to promote tumor cell growth [18], to mediate endotoxin-induced cardiomyocyte dysfunction [30], to activate NK cells [31] and to aggravate post-ischemic heart failure [32] through the activation of RAGE-dependent signaling. Therefore, we investigated whether RAGE ligation was responsible for the inhibitory effect of S100A8 in the PDGF-induced proliferation in ASMCs and found that the inhibition of proliferation by S100A8 can be partially abolished when ASMCs were treated with a neutralizing RAGE-blocking antibody. However, it was also found that a remaining anti-proliferation effect of S100A8 protein persisted after RAGE blocking (Fig. 3), indicating the inhibitory effect of S100A8 protein may not only be mediated by RAGE but also other cell-surface receptors possibly bind with S100A8 protein, such as Toll like receptor 4 and scavenger receptor CD36 [33]. Nevertheless, our results suggest that the cell membrane receptor RAGE appears to (at least partially) mediate the inhibitory effect of exogenous S100A8 protein in the PDGF-induced ASMCs proliferation.

## Conclusions

In summary, our study demonstrates that exogenous S100A8 protein can inhibit the PDGF-induced proliferation of ASMCs. This functional effect is dependent on the multi-ligand cell membrane receptor RAGE. In light of these new findings, together with the previous observation that S100A8 inhibits the PDGF-induced migration of ASMCs [10], S100A8 may become a potential therapeutic candidate to reduce and reverse the development of airway remodeling in chronic respiratory diseases.

## Abbreviations

ASMCs: airway smooth muscle cells
PDGF: platelet-derived growth factor
RAGE: the receptor for advanced glycation end-products
DMEM: Dulbecco’s modified Eagle’s medium
FBS: fetal bovine serum
